# Synthesis, Characterization and Molecular Modeling of Novel Oxoethyl methacrylate Polymers

**DOI:** 10.1021/acsomega.5c05759

**Published:** 2025-09-10

**Authors:** Seray Kayacık Bi̇li̇r, Nevin Çankaya, Mehmet Hanifi Kebi̇roğlu

**Affiliations:** † Graduate Education Institute, Department of Chemistry, 175652 Uşak University, Uşak 64200, Turkey; ‡ Vocational School of Health Services, Uşak University, Uşak 64200, Turkey; § Department of Medical Services and Techniques, Darende Bekir Ilıcak Vocational School, 531771 Malatya Turgut Özal University, 44700 Malatya, Turkey

## Abstract

The monomer 2-(2-methoxyphenylamino)-2-oxoethyl methacrylate (2MPAEMA) was synthesized and polymerized for the first time into its homopolymer and a copolymer with methyl methacrylate via free-radical polymerization. Structural verification was conducted using FT-IR and NMR spectroscopy, while thermal analysis confirmed the two-stage decomposition of both polymers. Quantum chemical calculations at the B3LYP/LanL2DZ level supported the experimental data, revealing significant intramolecular interactions, electronic delocalization, and thermal stability. The homopolymer exhibited a narrower HOMO–LUMO gap (4.954 eV) than the copolymer (5.207 eV), implying enhanced charge-transport potential. Molecular Electrostatic Potential and Density of States analyses further confirmed well-defined charge distribution and greater orbital overlap in the homopolymer. These results provide new insights into the structure–property relationships of 2MPAEMA-based polymers, highlighting their potential for optoelectronic, sensing, and thermoresponsive applications. Future studies will explore their biological activity and functional performance in targeted environments. FT-IR/NMR, thermal analysis, and DFT collectively indicate that 2MPAEMA polymers are wide-band gap and thermally robust, suggesting their suitability as dielectric matrices or UV-absorbing hosts.

## Graphical abstract

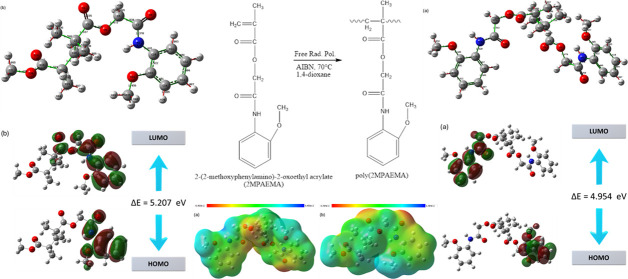

## 1 Introduction

Today, a large number of commercial acrylates and their derivatives are the focus of researchers due to their wide range of biomedical and industrial applications. The electron-attracting carbonyl functions in the vinyl group of acrylate monomers provide high reactivity in radical polymerization processes and allow flexibility in chain design. This chemical flexibility makes it possible to organize individual chain structures in homopolymer, block copolymer or random copolymer sequences to improve targeted mechanical, optical and biological performances. Besides biomedical uses such as tissue engineering, controlled drug release, contact lenses and bone cement, acrylates are also important in microscope slides, cosmetics, orthopedic materials, paints, coatings, adhesives and textiles.^1^ This diversity increases the interest in synthesizing new acrylate monomers and polymers and evaluating their biological effects. The biological activity of acrylates is particularly associated with the ability of prolonged release at low doses provided by amide and ester bonds. In recent years, interest in wound healing with phenyl acrylate-based polymers has also increased.^2^ Polymer microarray studies have revealed a copolymer of ethylene glycol dicyclopentenyl ether acrylate and di­(ethylene glycol) methyl ether methacrylate with antibacterial/antibiofilm properties, and the antioxidant potential of some acrylate derivatives has also been reported.^3^ Polymers produced from hydrophilic acrylic, methacrylic acid and acrylamide derivatives can support hydrogel systems widely used in agriculture.^4^

The growing world population increases yield pressure in crop production, while fungal and bacterial diseases pose a serious threat. Since the irregular use of existing antifungal agents can damage the ecosystem, the development of nontoxic and environmentally friendly antimicrobial acrylate compounds is a critical need.^1^

Therefore, synthesis of new acrylates, detailed investigation of their structure–property-performance relationships and multiscale evaluation of their biological activity are important for both sustainable materials engineering and global health and food safety.^5^ As evidenced in related studies, frontier-orbital analysis (HOMO–LUMO gap) and DOS/PDOS profiles offer a robust framework to discuss electronic stability, charge-transfer propensity, and optical activity, which motivates our use of these descriptors throughout this work.^6^ Recent enzymatic studies of acrylate polymerization, such as those using bacterial ester hydrolases, have highlighted the critical role of functional group orientation and active-site stabilization in guiding polymer growth, offering complementary mechanistic insight into synthetic approaches^7^ Beyond poly­(methyl methacrylate), functional methacrylates bearing hydroxyl (HEMA), epoxide (GMA), and tertiary-amine groups (DMAEMA) have been widely investigated as homopolymers and MMA-copolymers for coatings, optics, and biointerfaces. These systems show how pendant functionality modulates thermal stability (onset and multistep degradation), segmental dynamics (chain stiffening/softening), intermolecular interactions (H-bonding, Lewis’s acid base, π–π contacts), and, when assessed computationally, frontier-orbital trends that correlate with dielectric/optical behavior. For instance, MMA-*co*-DMAEMA introduces basic sites that elevate intermolecular cohesion and can shift Tmax;^8^ MMA-*co*-HEMA adds protic H-bond donors/acceptors that reshape glass transition and decomposition profiles;^9^ GMA-containing copolymers increase cross-linkability and thus thermal resistance.^10^ In aromatic methacrylates, pendant rings frequently promote intrachain stacking and broaden DOS features, even when global band gaps remain wide.^11^ This body of work provides a benchmark for interpreting how our amide- and anisole-substituted oxoethyl-methacrylate segments influence spectroscopy, thermal response, and electronic descriptors in both homopolymer and MMA-copolymer forms.

In our previous studies, we performed the synthesis and characterization of 2-(2-methoxyphenylamino)-2-oxoethyl methacrylate (2MPAEMA) experimentally and theoretically and reported the biological, experimental, antimicrobial, antibiofilm, and antioxidant effects of this molecule.^5^ In this study, we synthesized and characterized the homopolymer of 2MPAEMA and its copolymer with methyl methacrylate, which is not available in the literature. The aim of this research is to provide the first comprehensive evaluation of the structure–property–activity relationships of the newly synthesized 2MPAEMA homopolymer and its methyl-methacrylate copolymer through integrated spectroscopic, thermal, theoretical, and chemical analyses.^12^ For this we used advanced computational methods, particularly DFT, to obtain highly accurate predictions of the molecule’s properties. LanL2DZ is more commonly used for heavier atoms; however, we selected it due to (i) its historical use in similar polymer studies, (ii) its favorable balance between cost and performance for large systems, and (iii) the excellent agreement of calculated results with experimental data (IR/NMR/thermal). To improve clarity, we now specify that trimeric oligomers capped with methyl groups were used to simulate local polymer structure. These segments effectively capture repeating intramolecular interactions and were validated through close matching with observed thermal, spectroscopic, and electronic data, making them suitable representatives of bulk-like behavior. Thus, our work not only characterizes polymers containing 2MPAEMA, but also suggests future research directions to further explore and optimize their properties while identifying their potential applications in other applications. These contributions position our work as a significant advance in the field, providing valuable insights and a foundation for future research.^13^

## 2 Experimental Section

### 2.1 Materials

For monomer synthesis, 4-methoxyaniline, triethylamine, chloroacetyl chloride, and sodium acrylate (Aldrich) were used as received. Acetone, acetonitrile, and 1,4 dioxane solutions were dried on anhydrous MgSO_4_ and freshly distilled before use.^5^ For polymer synthesis, azobis­(isobutyronitrile) was a radical initiator, 1,4-dioxane as a solvent and ethyl alcohol as a precipitant. The methyl methacrylate (MMA) monomer used in the copolymer synthesis was reacted by removing the hydroquinone that inhibits polymerization in it.^14^

### 2.2 Equipment

The FTIR spectra of all samples were performed with a PerkinElmer Spectrum Two (UATR) IR spectrometer in the range of 4000–450 cm^–1^. ^1^H and ^13^C NMR measurements were conducted on Bruker TopSpin Ultra Shield 400 MHz in Chloroform-d as solvent. Thermal analyze of the polymer were obtained with a Hitachi 7000 TGA/DTA/DTG (Thermal Gravimetric Analysis/Differential Thermal Analysis/Differential Thermogravimetric Analysis) simultaneous system a heating rate of 10 °C min^–1^ in nitrogen atmosphere, from room temperature to 600 °C temperatures.

### 2.3 Synthesis of 2MPAEMA Homopolymer (Poly­(2MPAEMA))

2MPAEMA monomer was resynthesized.^5^ For the synthesis of homopolymer, 2MPAEMA (1 mmol) monomer was dissolved in 1,4-dioxane solvent, and polymerized with 1% azobis­(isobutyronitrile) radical initiator and nitrogen as an inert gas at 70 °C for 36 h. The synthesized homopolymer was crystallized to remove impurities with ethyl alcohol (yield 80%). The chemical structure of homopolymer was characterized by spectroscopic methods by FTIR and ^1^H and ^13^C NMR. The synthesis of the homopolymer of 2MPAEMA is shown in Figure 1.

**Figure 1:**
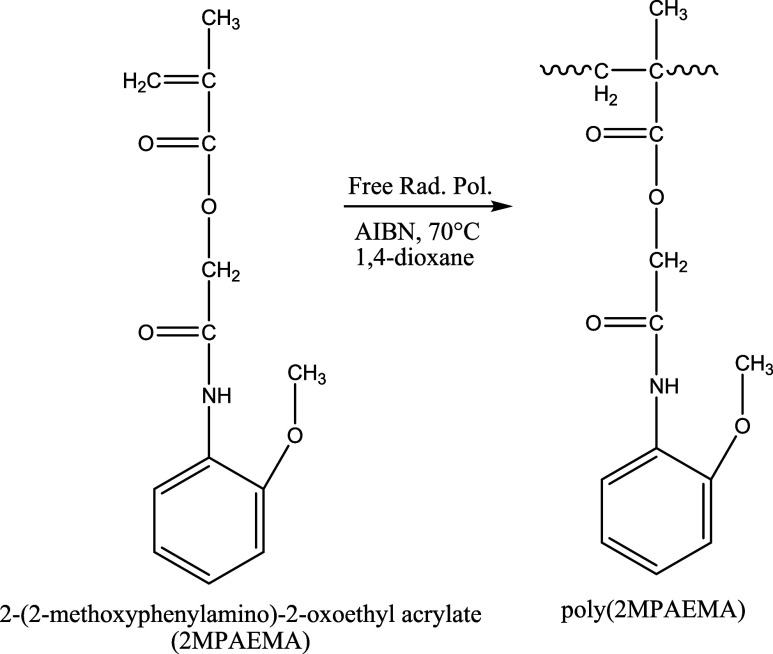
Synthesis scheme of poly­(2MPAEMA) homopolymer.

### 2.4 Synthesis of Copolymerization of Methyl Methacrylate with 2MPAEMA (2MPAEMA-*co*-MMA)

The copolymerization of methyl methacrylate (MMA) and 2MPAEMA was synthesized similar to the homopolymer synthesis. Two suitable monomers 2MPAEMA (1 mmol) and MMA (1 mmol) were dissolved in 1,4-dioxane solvent, and polymerized with 1% azobis­(isobutyronitrile) radical initiator and nitrogen as an inert gas at 70 °C for 36 h. The resulting copolymer was crystallized to remove impurities with ethyl alcohol (yield 85%). Synthesis of copolymer of 2MPAEMA and MMA (2MPAEMA-*co*-MMA) is shown in Figure 2. The chemical structure of 2MPAEMA-*co*-MMA was characterized by spectroscopic methods by FTIR, ^1^H and ^13^C NMR.

**Figure 2:**
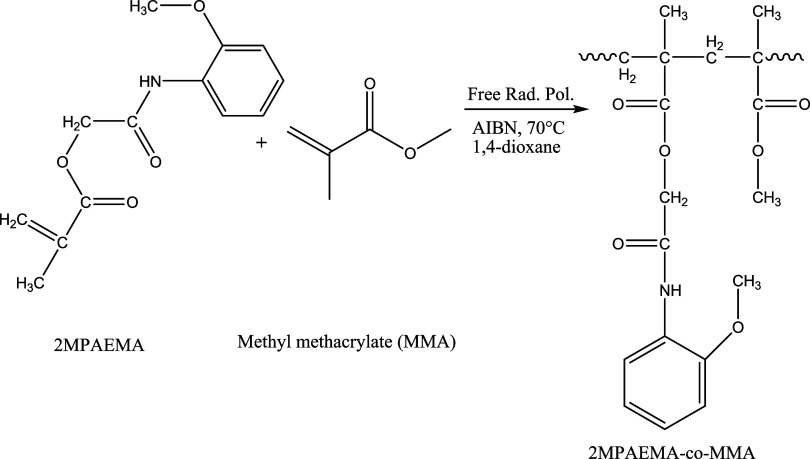
Synthesis scheme of 2MPAEMA-*co*-MMA copolymer.

### 2.5 Calculation Details

Recent studies have demonstrated the power of combining DFT-based quantum chemical calculations with molecular docking and dynamics to reveal structural stability, electronic behavior, and biological potential of complex systems, offering a robust pathway for designing multifunctional materials.^15^ All quantum-chemical calculations were performed with Gaussian 16. Geometry optimizations and harmonic frequency calculations were carried out at the DFT/B3LYP level with the LanL2DZ basis set. While the LANL2DZ basis set was selected for its favorable balance between accuracy and computational cost, particularly in polymer systems, future studies employing all-electron basis sets such as 6–31G­(d,p) could provide additional cross-validation of the present electronic structure predictions. All optimized structures were confirmed as true minima by the absence of imaginary frequencies. Natural Bond Orbital (NBO) analyses were performed using Gaussian 16. Density of states (DOS), conceptual-DFT descriptors and reduced density gradient (RDG/NCI) analyses were obtained with Multiwfn 3.8, and visualizations were produced with VMD 1.9.4 and GaussView 6.0.^16^ The vibrational response of 2MPAEMA-*co*-MMA and poly­(2MPAEMA) was characterized through experimental spectroscopy and quantum-chemical simulation.^5^ To assess their electronic architecture and conformational resilience, we ran DFT/B3LYP calculations with the LanL2DZ basis set on both the 2-(2-methoxyphenylamino)-2-oxoethyl methacrylate (2MPAEMA) monomer and its homopolymer.^17^

In our DFT calculations, trimeric oligomers consisting of three repeating units and capped with methyl groups at both chain ends were the representative model. This chain length was chosen as it effectively captures the key intramolecular interactions and conformational features of the bulk polymer while keeping computational costs manageable.^18^ Electronic structure methods can provide similar qualitative trends; among those commonly used for polymers, the B3LYP/LanL2DZ level gave results in good agreement with our experimental data. Therefore, for clarity and brevity, we report and discuss only the DFT results, acknowledging the approximate nature of DFT and its dependence on the chosen exchange-correlation function.^19^ Geometry optimizations converged to true minima, as evidenced by the absence of imaginary frequencies in subsequent frequency analyses.^20^ The predicted bond lengths, bond angles, and dihedral parameters closely matched values reported for analogous acrylate derivatives, supporting the reliability of the chosen computational protocol.^21^ Natural Bond Orbital (NBO) analysis further elucidated intramolecular electron delocalization, charge-transfer pathways, and bonding characteristics within both 2MPAEMA-*co*-MMA and poly­(2MPAEMA).^22^ The spatial arrangement of donor and acceptor atoms suggests a dynamic hydrogen-bonding network, which may be advantageous in applications requiring environmental responsiveness, such as drug delivery or smart polymer systems.^23^ Collectively, these findings provide a robust platform for deeper exploration of the materials’ electronic and structural behavior. The primary energetic stabilization in a bimolecular reaction often stems from the overlap between the HOMO of one reactant and the LUMO of the other, a phenomenon known as a frontier-molecular-orbital (FMO) interaction.^24^ The governing equations used for Frontier Molecular Orbital (FMO) analysis are presented in the following section.^25^ These equations represent the global reactivity descriptors derived from conceptual Density Functional Theory (DFT), commonly used to characterize electronic stability, reactivity, and charge-transfer potential.^26^ These include ionization potential (I), electron affinity (A), chemical hardness (η), softness (σ), electronegativity (χ), chemical potential (μ), and global electrophilicity index (ω).^27^ These descriptors are calculated based on HOMO and LUMO energies and provide insights into how the molecule will behave in electronic excitation or charge transport scenarios.^28^

1 $$I=-E_{HOMO}$$

2 $$A=-E_{LUMO}$$

3 $$\eta =\frac{1}{2}{\left[\frac{\partial^{2}E}{\partial^{2}N}\right]}_{v\left(r\right)}=\frac{I-A}{2}$$

4 $$\left\langle \alpha \right\rangle =\frac{1}{3}\left[\alpha_{\mathrm{xx}}+\alpha_{\mathrm{yy}}+\alpha_{\mathrm{zz}}\right]=\sigma =\frac{1}{\eta }$$

5 $$\mu =-\chi ={\left[\frac{\partial E}{\partial N}\right]}_{V\left(r\right)}=-\left(\frac{I+A}{2}\right)$$

6 $$\omega =\frac{\chi^{2}}{2\eta }$$

7 $$\varepsilon =\frac{1}{\omega }$$

8 $$\omega^{+}=\frac{{\left(I+3A\right)}^{2}}{16\left(I-A\right)}$$

9 $$\omega^{-}=\frac{{\left(3I+A\right)}^{2}}{16\left(I-A\right)}$$

10 $$\mathrm{MAE}=\frac{1}{n}\sum_{i=1}^{n}\left|x_{i}^{\exp}-x_{i}^{\mathrm{theo}}\right|$$

11 $$\mathrm{RMSE}=\sqrt{\frac{1}{n}\sum_{i=1}^{n}{\left(x_{i}^{\exp}-x_{i}^{\mathrm{theo}}\right)}^{2}}$$

12 $$R^{2}=1-\frac{\sum_{i=1}^{n}{\left(x_{i}^{\exp}-x_{i}^{\mathrm{theo}}\right)}^{2}}{\sum_{i=1}^{n}{\left(x_{i}^{\exp}-x_{i}^{-\exp}\right)}^{2}}$$

Statistical equations were used to quantify the agreement between experimental and theoretical spectroscopic values, and the calculated results are summarized in Table 2-spectroscopic characterization section.^29^

These parameters collectively define the electronic character of the material. For example, a smaller HOMO–LUMO gap (Δ*E*) suggests enhanced charge mobility and lower excitation energy, while higher electrophilicity (ω) reflects greater tendency to accept electrons. Softness and hardness describe the molecule’s polarizability and resistance to deformation. Together, these descriptors help interpret the material’s potential performance in specialized optoelectronic applications such as UV photodetectors and dielectric components, as well as in sensing and thermoresponsive systems.^30^ The statistical equations were used to quantify the agreement between experimental and theoretical spectroscopic values, and the calculated results are presented in the spectroscopic characterization section.

### 2.6 Theoretical Spectroscopic Analysis

The harmonic vibrational frequencies of the 2MPAEMA-*co*-MMA and poly­(2MPAEMA) molecular structures were calculated using Density Functional Theory (DFT) at the B3LYP level of theory with the LanL2DZ basis set.^31^ The vibrational analysis confirmed the absence of imaginary frequencies, indicating that the optimized molecular geometries correspond to true minima on the potential energy surface, and therefore the structural stability of these polymer systems was validated.^32^ Vibrational spectroscopic analysis pinpointed the characteristic IR absorption maxima corresponding to molecular vibrational modes. In addition to vibrational analysis, NMR chemical shifts were calculated using the GIAO method, and theoretical spectra were plotted to support experimental findings.^33^ In addition to vibrational analysis, the molecular geometries of the studied systems were initially refined through comprehensive NMR calculations performed at the same theoretical level (B3LYP).^34^ Since NMR computations allow the simultaneous observation of proton-bearing sites and their neighboring functional groups, they serve as a powerful tool for elucidating the detailed architecture of the molecule under investigation.^35^ Using the GIAO method, the chemical shifts were then calculated and the theoretical NMR spectrum plotted.^36^

### 2.7 Molecular Electrostatic Potential (MEP)

To explore the electronic charge distribution and identify reactive regions within the 2MPAEMA-*co*-MMA and poly­(2MPAEMA) molecular structures, MEP analysis was conducted.^37^ Utilizing optimized molecular geometries obtained from DFT calculations implemented in Gaussian 16, the MEP surface was calculated.^38^ This computational method involves projecting electrostatic potential data onto the electron density contour to highlight electron-rich and electron-poor areas.^39^ MEP analysis was performed to explore the electronic charge distribution and identify reactive regions within the molecular structures. The MEP surface was mapped onto the electron density to highlight electron-rich and electron-poor areas that indicate potential sites for nucleophilic and electrophilic interactions.^40^

### 2.8 Thermochemistry Surface Maps (TCSM)

Thermodynamic stability and spatial energy distribution were evaluated using Thermochemistry Surface Maps (TCSM). Vibrational frequency data were used to calculate thermal energy, heat capacity, and entropy. The results were visualized as thermochemical indices projected onto electron-density isosurfaces.^41^ To confirm that the optimized molecular geometries corresponded to genuine minima on their respective potential energy surfaces, vibrational frequency analyses were carried out.^42^ These thermochemical maps provide valuable insights into the localized thermodynamic behavior, elucidating molecular stability and identifying potential energetic pathways relevant to the performance of 2MPAEMA-*co*-MMA and poly­(2MPAEMA) under diverse environmental conditions. The computations were conducted at standard ambient conditions of 298.15 K temperature and 1 atm pressure.^43^ Thermochemical surface maps were designed to probe each molecule’s thermodynamic stability and energy distribution.^44^ TCSM were generated by distributing vibrational thermochemical functions over atoms and projecting the resulting scalar field onto the 0.001 au electron-density isosurface.^45^ Specifically, from the RRHO analysis we obtained the mode-resolved thermal energy and heat capacity; per-atom contributions were estimated using normalized eigenvector participation factors for each mode, and then summed at 298.15 K and 1 atm.^46^ The resulting dimensionless local thermochemical index was min–max normalized to [0, 1] before visualization (deep-red = lower local thermochemical energy density, i.e., stabilizing; yellow/orange = higher, i.e., destabilizing).^47^

### 2.9 Density of States (DOS)

First-principles DFT methods provide an efficient way to analyze and, when necessary, tailor the electronic DOS of metallic and polymeric frameworks.^48^ The electronic profiles of 2MPAEMA-*co*-MMA and poly­(2MPAEMA) were analyzed using DOS calculations based on the previously optimized structures.^35^ Total and projected DOS plots provided insight into the HOMO–LUMO gap, state density, and electronic structure.^49^ Inspection of the DOS landscapes indicates a gradual relocation of electronic density from the outer moieties toward the molecular core, a behavior commonly noted in related macromolecular systems.^50^ For partially filled-band materials, the calculated DOS approaches zero at the Fermi level, in line with theoretical expectations.^51^ The DOS furnishes a quantitative picture of how closely electronic states are spaced that is, how densely electrons are packed within a quantum system.^52^

### 2.10 Non-Covalent Interactions (NCI)

Reduced Density Gradient (RDG) and NonCovalent Interaction (NCI) analysis were used to map noncovalent interactions within the polymer structures.^53^ Isosurfaces and scatter plots identified attractive hydrogen bonds, dispersive van der Waals interactions, and steric repulsions, providing a spatial and energetic picture of weak interactions contributing to structural stability.^54^ In these plots, negative sign­(λ_2_)­ρ values identify attractive regions (e.g., hydrogen bonds), values near zero denote dispersive van der Waals contacts, and positive values reveal steric repulsion.^55^ Numerical evaluations were carried out with Multiwfn, while visual renderings were generated in VMD and GaussView, yielding a spatially resolved picture of the interaction landscape and its contribution to molecular stability.^56^ More broadly, the NCI framework offers an advanced means of classifying and quantifying weak intermolecular forces, affording a nuanced perspective on molecular organization and reactivity.^57^

## 3 Result and Discussion

### 3.1 Spectroscopic Characterization

The calculated and experimental FT-IR and NMR results align closely, demonstrating strong mutual consistency.^58^ Theoretical values were calculated using DFT/B3LYP with the LanL2DZ basis set. To enhance clarity and allow direct comparison between the experimental and theoretical findings, we provide a summary Table 1 highlighting the observed and DFT-predicted FTIR and NMR values. The good agreement between these data sets supports the structural assignments and confirms the reliability of the computational model.^59^

**Table 1:** Comparison of Experimental and Theoretical FTIR and NMR Values for Poly­(2MPAEMA) and 2MPAEMA-*co*-MMA

	**poly(2MPAEMA)**		**2MPAEMA-** *co* **-MMA**		
**technique**	**exp. value**	**theoretical value**	**exp. value**	**theoretical value**	**assignment**
FTIR	1740 cm^–1^	1735 cm^–1^	1728 cm^–1^	1735 cm^–1^	ester carbonyl stretch
FTIR	1693 cm^–1^	1680 cm^–1^	1666 cm^–1^	1680 cm^–1^	amide carbonyl stretch
FTIR	3410 cm^–1^	3300 cm^–1^	3325 cm^–1^	3300 cm^–1^	amide N–H stretch
^1^H NMR	8.2 ppm	9 ppm	10.2 ppm	10 ppm	amide proton
^1^H NMR	6.3–6.9 ppm	6.5 ppm	6.6–7.8 ppm	7.5 ppm	aromatic protons
^1^H NMR	3.7 ppm	3.8 ppm	3.7 ppm	3.8 ppm	methoxy protons
^13^C NMR	175.4 ppm	175 ppm	178.9 ppm	180 ppm	ester carbonyl carbon
^13^C NMR	1740 cm^–1^	165 ppm	168.3 ppm	170 ppm	amide carbonyl carbon
^13^C NMR	1693 cm^–1^	115 ppm	110.5–148.9 ppm	145 ppm	aromatic ring carbons

In addition to the direct comparison, statistical parameters were calculated to evaluate the agreement between experimental and theoretical values. The mean absolute error (MAE), root-mean-square error (RMSE), and correlation coefficient (*R* ^2^) demonstrated an excellent fit (MAE = 14.9 and 5.9; RMSE = 37.0 and 9.9; *R* ^2^ = 0.9998 and 0.9999 for poly­(2MPAEMA) and 2MPAEMA-*co*-MMA, respectively), as summarized in Table 2.

**Table 2:** Statistical Measures of Agreement between Experimental and Theoretical Data

**polymer system**	**property**	**MAE**	**RMSE**	***R*** ^**2**^
poly(2MPAEMA)	FTIR + NMR combined	14.9	37.0	0.9998
2MPAEMA-*co*-MMA	FTIR + NMR combined	5.9	9.9	0.9999

#### 3.1.1 Spectroscopic Characterization of 2MPAEMA Homopolymer

The FTIR, ^1^H and ^13^C NMR spectra of the synthesized 2MPAEMA homopolymer are indicated in Figures 3 and 4. FTIR (cm^–1^, the most characteristic bands): 1740 (C=O ester stretch), 1693 (C=O amide stretch), 1602 (C=C stretch on aromatic ring), 3410 (N–H stretching), 2940 (C–H aliphatic stretch), 1253 and 1536 (C–O–C symmetric and asymmetric stretching) peaks. The absence of C=C olefinic stretch is the first evidence of homopolymer formation.^5^

**Figure 3:**
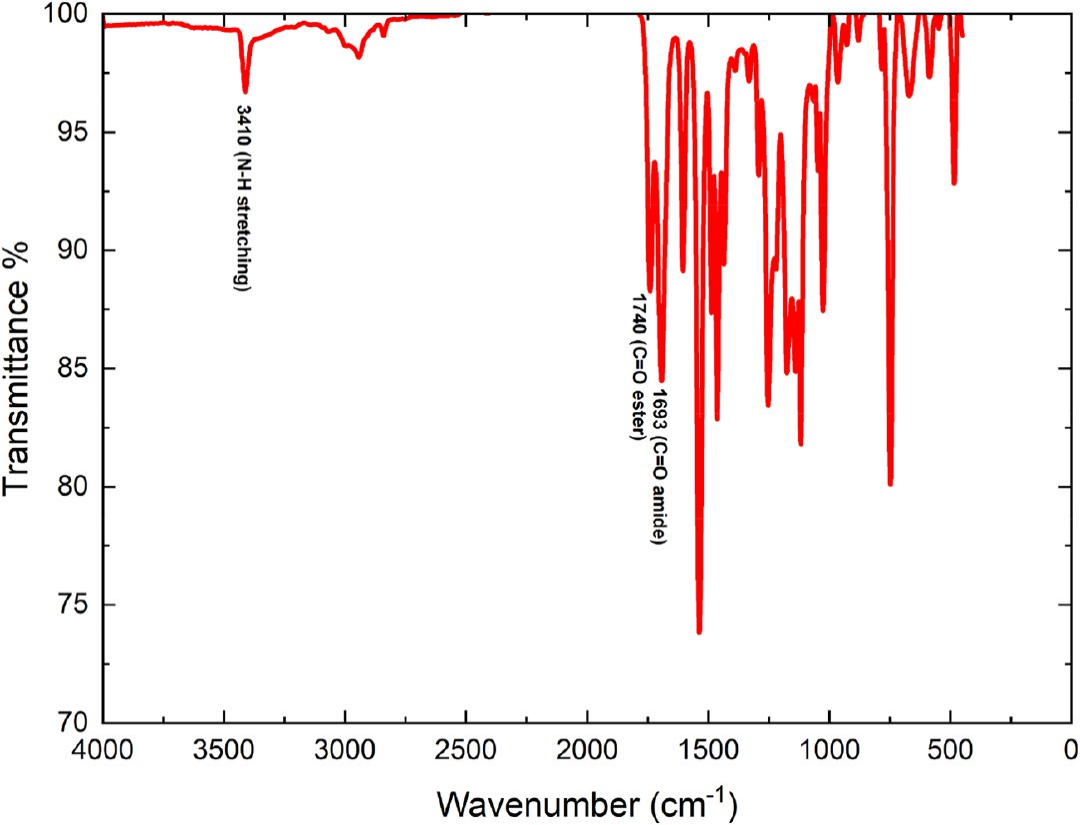
FTIR spectrum of the 2MPAEMA homopolymer.

**Figure 4:**
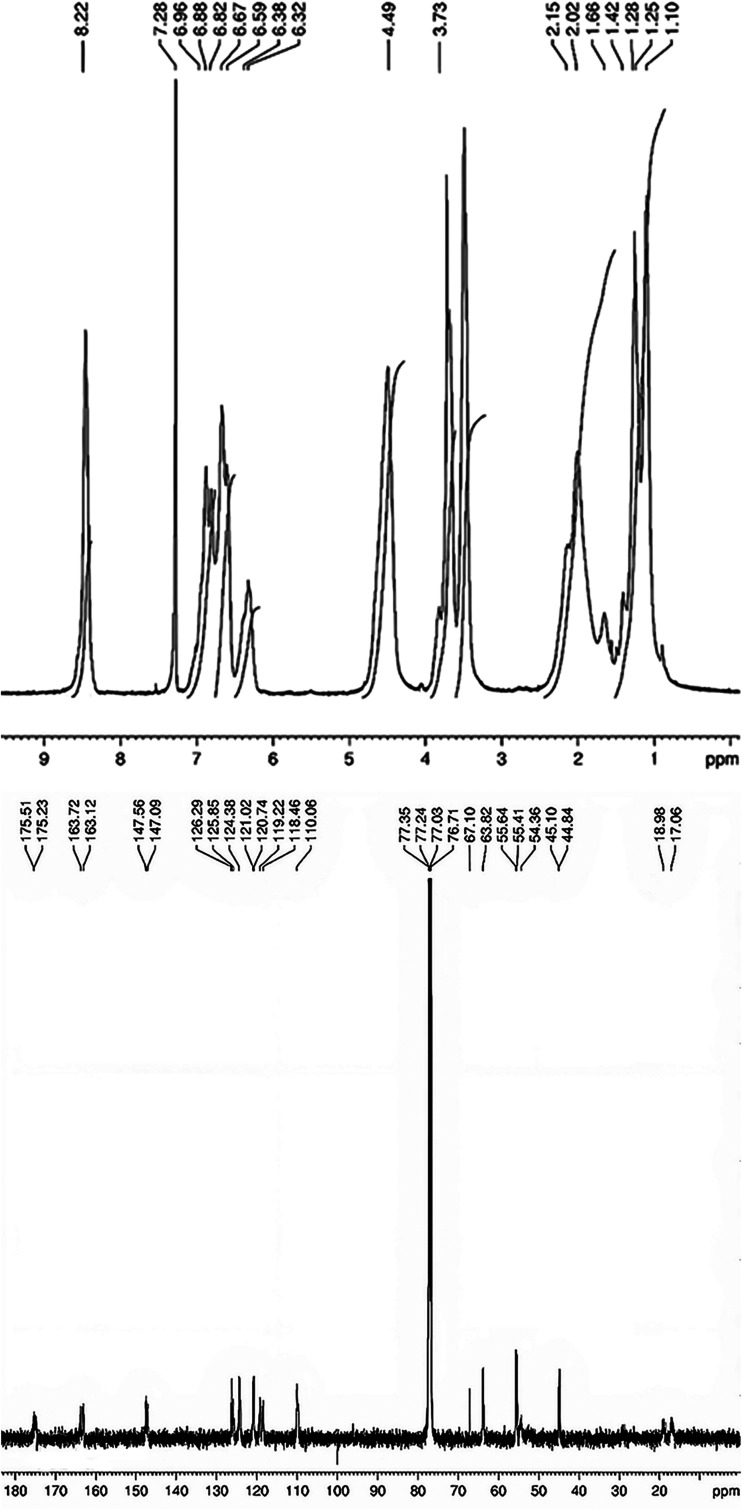
^1^H and ^13^C NMR spectra of the 2MPAEMA homopolymer.

^1^H NMR spectrum of homopolymer following peaks appear; at 7.3 ppm for Chloroform-d solvent protons; at 8.2 ppm for NH proton; at 6.9–6.3 ppm for aromatic ring protons; at 4.5 ppm for O–CH_2_ protons; at 3.7 ppm for aromatic O–CH_3_ protons; at 2.1 ppm for C–CH_3_ protons; 1.7–1.1 ppm for the polymer chain protons. ^13^C NMR spectrum of homopolymer following peaks appear; at average 77.1 ppm for Chloroform-d solvent protons, at 175.4 ppm for O=C-OCH_2_, at 163.4 ppm for O=C-NH, at 147.3 ppm for Ar­(C)-OCH_3_, at between 126.3–110.1 ppm for aromatic ring carbons, at 67.1 ppm for O–CH_2_–C=O, at 63.8 ppm for Ar–O–CH3, at 55.1 ppm for carbons in the polymer chain CH_3_–C, at 44.9 ppm for carbons in the polymer chain C–CH_2_, at 18.9 and 17.1 ppm for repeating carbons in the polymer chain.

#### 3.1.2 Spectroscopic Characterization of 2MPAEMA-*co*-MMA Copolymer

The FTIR, ^1^H and ^13^C NMR spectra of the synthesized 2MPAEMA-*co*-MMA copolymer are indicated in Figures 5 and 6. FTIR (cm^–1^, the most characteristic bands): 1728 (C=O ester stretch), 1666 (C=O amide stretch), 1605, (C=C stretch on aromatic ring), 3325 (N–H broader peak stretching), 1242 and 1512 (C–O–C symmetric and asymmetric stretching).

**Figure 5:**
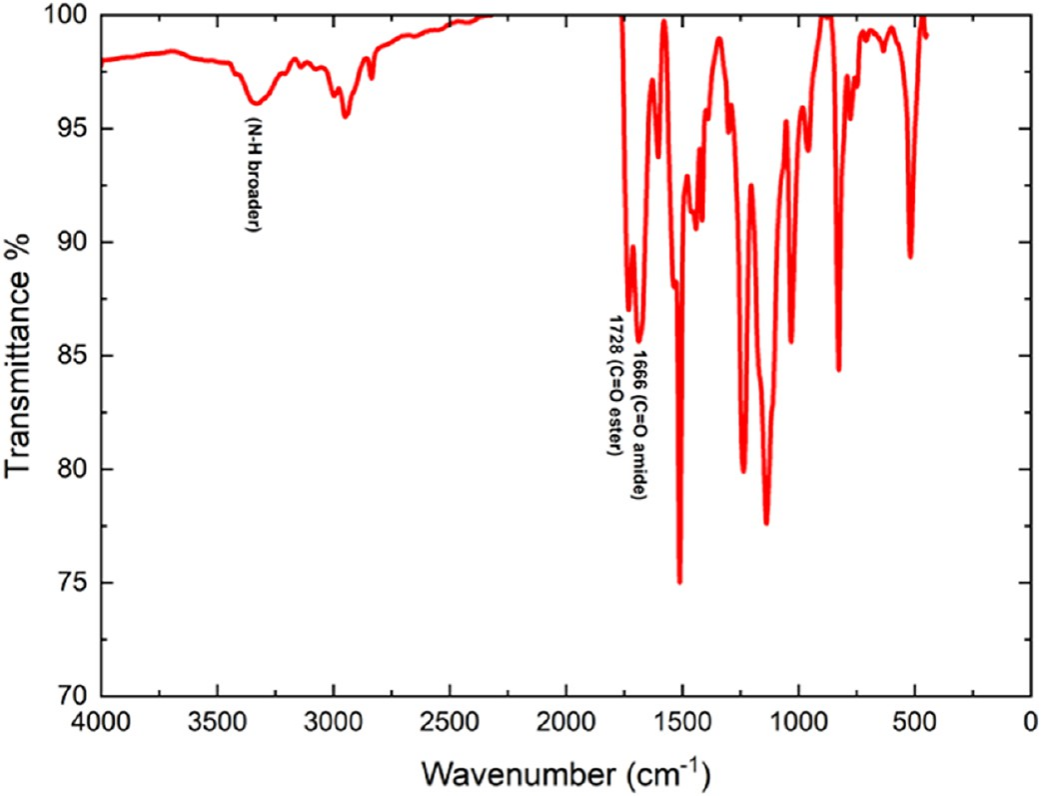
FTIR spectrum of the 2MPAEMA-*co*-MMA copolymer.

**Figure 6:**
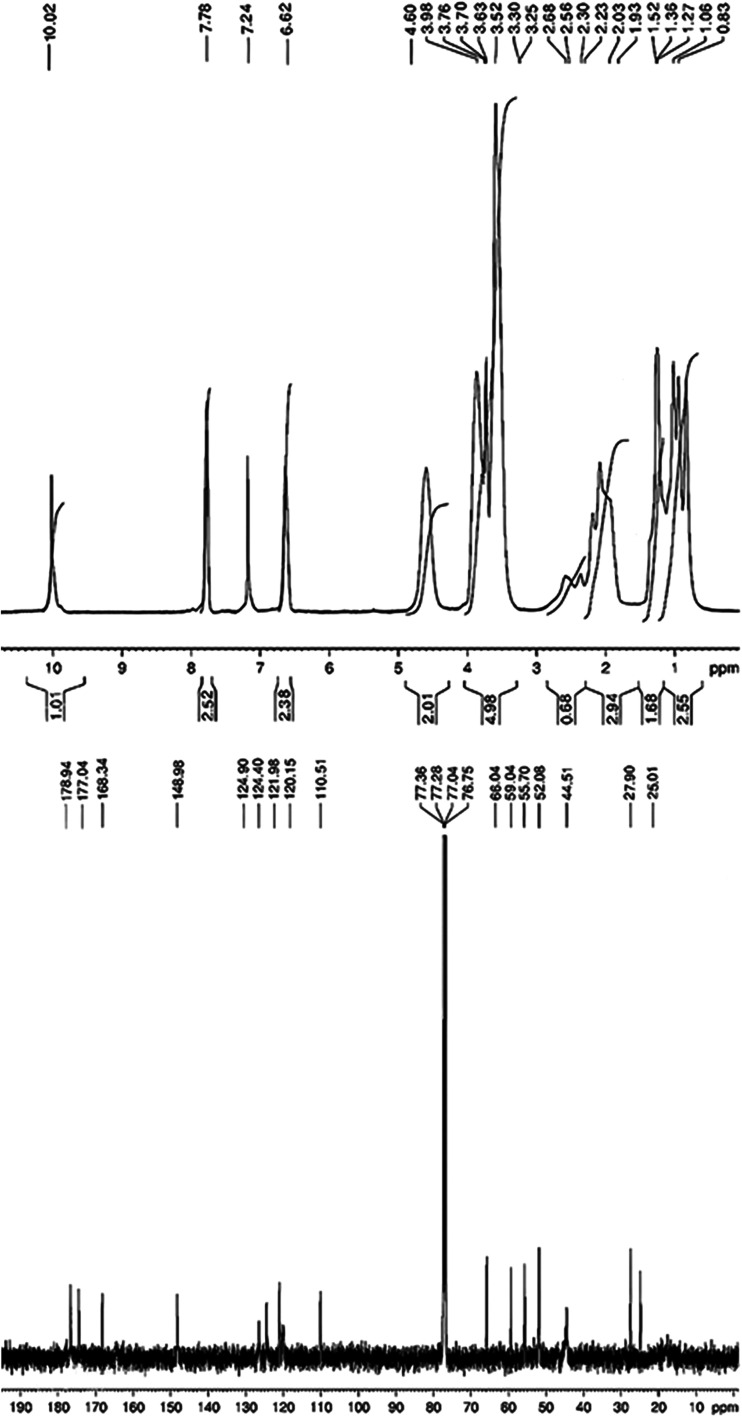
^1^H and ^13^C NMR spectra of the 2MPAEMA-*co*-MMA copolymer.

^1^H NMR spectrum of copolymer following peaks appear; at 7.2 ppm for Chloroform-d solvent protons; at 10.2 ppm for NH proton, at 7.8 and 6.6 ppm for ring protons, at 4.6 ppm for O–CH_2_–C protons, at 3.7 ppm for aromatic O–CH_3_ protons, at 3.6 ppm for O–CH_3_ protons, at 2.6–2.4 ppm for C–CH_2_–C protons, at 2.2–2.0 ppm of C–CH_3_ protons, at 1.5–0.8 ppm for protons in the repeating polymer unit. ^13^C NMR spectrum of copolymer following peaks appear; at average 77.1 ppm for d-chloroform solvent protons; at 178.9 ppm for O=C–O–, at 177.0 ppm for O=C–O–CH_3_, at 168.3 ppm for O=C-NH_2_, at 148.9 ppm for Ar­(C)-OCH_3_, at 124.9, 124.4, 121.9, 120.2, 110.5 ppm for aromatic ring carbons, at 66.0 ppm for O–CH_2_, at 59.0 ppm for C–CH_2_–C, at 55.7 ppm Ar-OCH_3_, at 52.1 ppm for O=C–O–CH_3_, at 44.5 ppm for some carbons in the polymer chains, at 27.9 ppm for C–CH_3_ belonging to 2MPAEMA, at 25.0 ppm for C–CH_3_ belonging to MMA and some polymer chain carbons.

### 3.2 Thermal Characterization of Homo and Copolymer

Thermal analysis methods help determining the thermal stabilities of polymers and provide information about their thermal behavior.^60^ The decomposition temperature and the temperature at weight loss are taken as a measure of thermal stability. The thermal properties of polymers were determined by TGA/DTA/DTG simultaneous system. Some basic thermal data such as decomposition temperatures and mass loss percentages at various temperature ranges were calculated. The degradation of both polymers from the thermogram was observed at two level. Some important thermal results for the homopolymer are From the DTG curve, it was seen that the maximum decomposition temperature was approximately 363 and 412 °C. Decomposition temperature at 20, 25, and 50% is 321, 333 and 364 °C; weight loss at 400 and 500 °C is 79 and 96%; residue at 600 °C is 3%, respectively. Some important thermal results for the copolymer are From the DTA curve, it was seen that the initial decomposition temperature was approximately 300 °C. From the DTG curve, it was seen that the maximum decomposition temperature was approximately 373 and 440 °C. Decomposition temperature at 10%, and 50% is 308 °C, and 374 °C; weight loss at 400, 450, and 500 °C is 65, 89, and 94% respectively, residue at 600 °C is 6%. The thermal curves of the polymers are given in Figure 7. In Figure 7, the axes for thermal analysis curves now indicate Temperature (°C) on the *x*-axis and Weight (%) or Heat Flow (μV) on the *y*-axis, depending on the curve type (TGA, DTG, or DTA).

**Figure 7:**
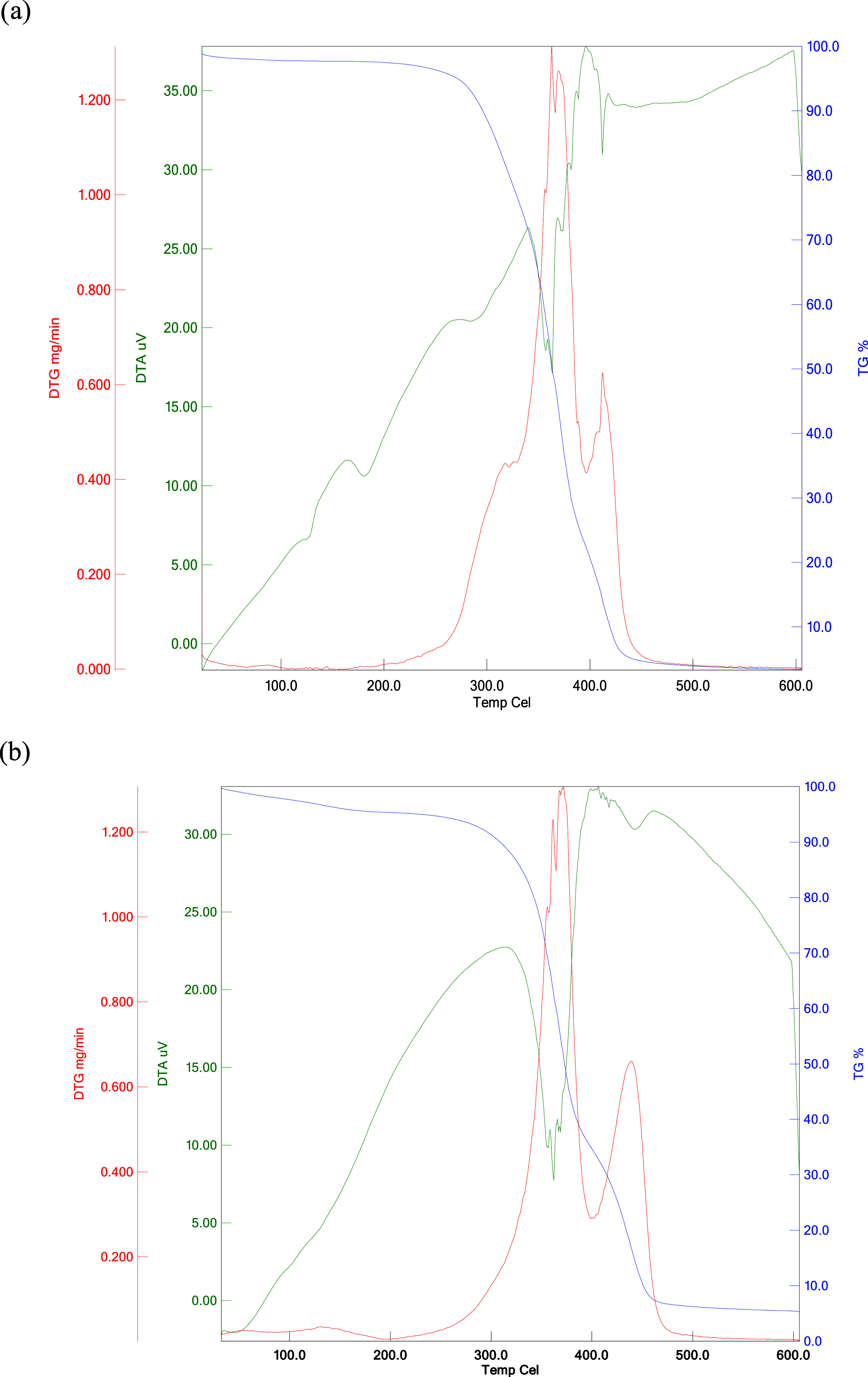
TGA/DTA/DTG Thermal curves of (a) poly­(2MPAEMA) and (b) 2MPAEMA-*co*-MMA.

### 3.3 Geometry Optimization and Natural Bond Orbital (NBO) Analysis

Figure 8 illustrates the optimized molecular geometries and Natural Bond Orbital (NBO) interactions of homo and copolymer, obtained using DFT calculations at the B3LYP level of theory using the LanL2DZ basis set. Molecular geometry optimizations were carried out using DFT at the B3LYP level of theory with the LanL2DZ basis set.^5^ In poly­(2MPAEMA) (Figure 8a), multiple intramolecular hydrogen bonds are observed across adjacent repeating units, indicating increased conformational freedom. The NBO analysis demonstrates prominent hyperconjugative interactions, particularly between nitrogen lone pairs and the antibonding orbitals of adjacent carbonyl groups, which play a critical role in stabilizing the extended polymer chain.^61^ For 2MPAEMA-*co*-MMA, significant intramolecular hydrogen bonding interactions between hydroxyl, carbonyl, and amine functionalities were identified through NBO analysis, suggesting a stabilized semirigid conformation (Figure 8b).^62^ Including the MMA unit introduces additional steric constraints, contributing to a more compact and less flexible structure.

**Figure 8:**
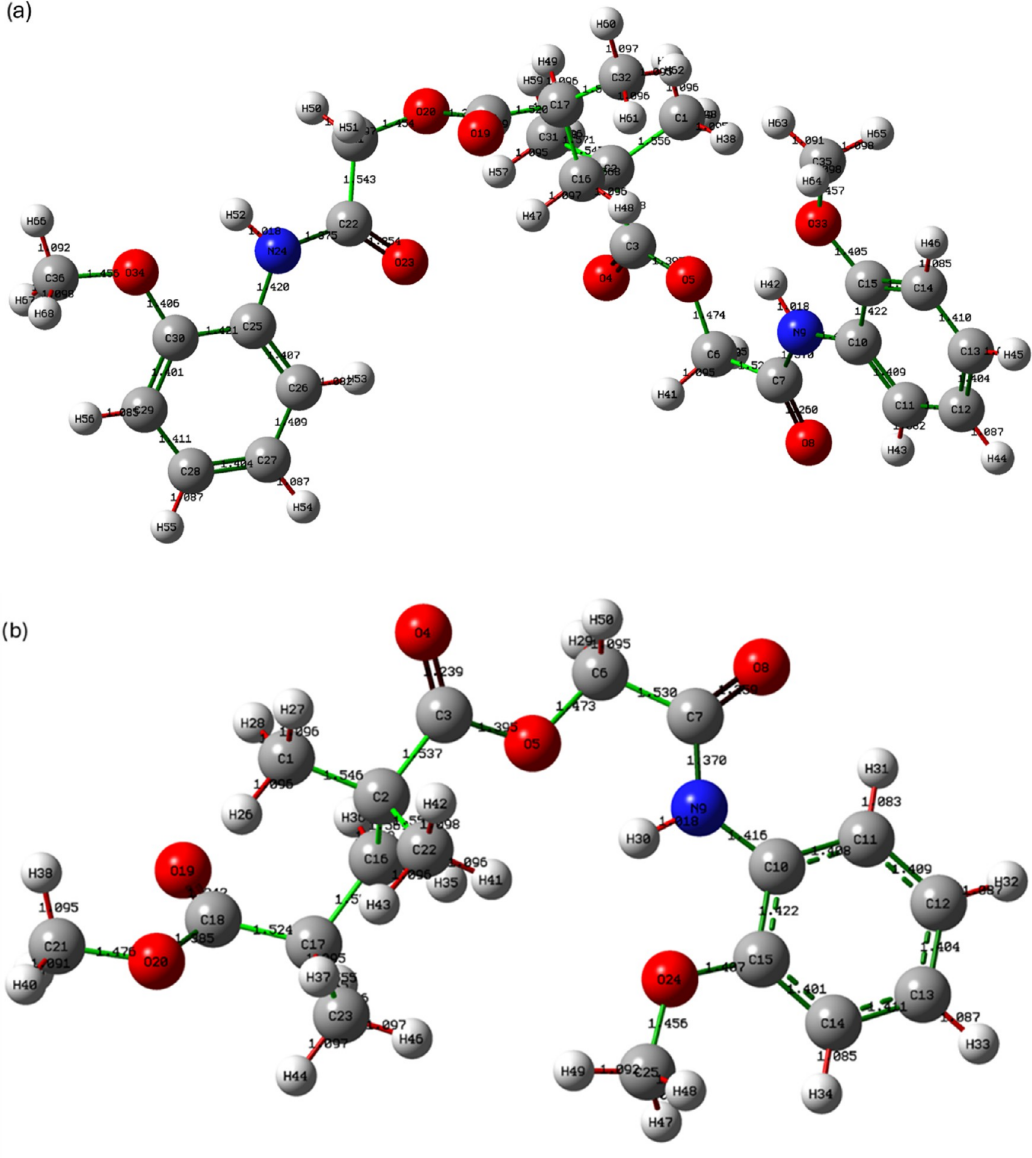
Geometry Optimization and Natural Bond Orbital (NBO) Analysis of (a) poly­(2MPAEMA) and (b) 2MPAEMA-*co*-MMA.

NBO analysis highlighted significant charge-transfer pathways, particularly LP­(O)→π*­(C=O) and LP­(N)→σ*­(C–O) interactions, which stabilize the polymer backbone. For poly­(2MPAEMA), the strongest LP­(O_amide)→π*­(C=O) delocalizations reached up to ∼62.5 kcal·mol^–1^, supporting intrachain stabilization. In the copolymer, additional LP­(O_ester)→π*­(C=O_MMA) interactions (E(2) ≈ 29.4 kcal·mol^–1^) were observed, indicating local conformational locking induced by the MMA unit. These results confirm that NBO delocalization effects contribute significantly to the observed conformational stability and complement the hydrogen-bonding interactions shown in the Figure 9.

**Figure 9:**
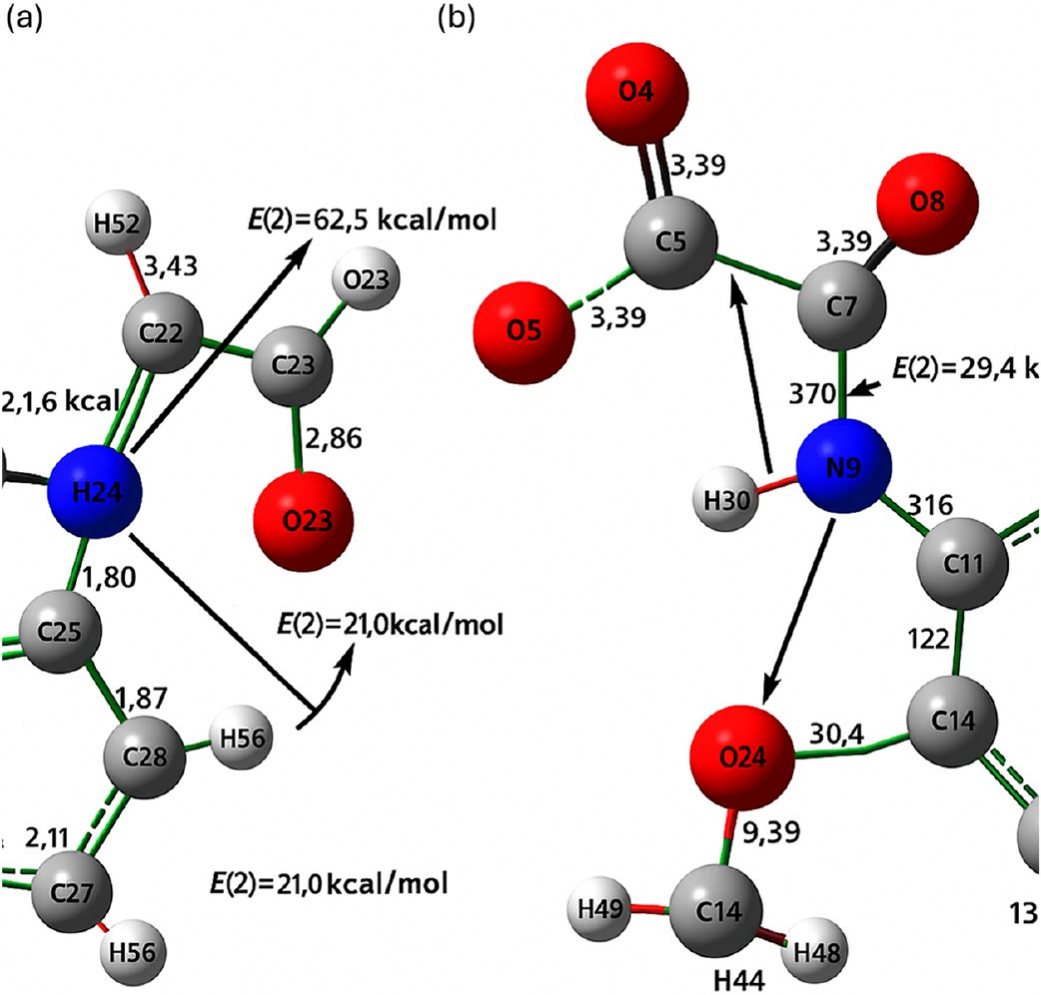
Optimized geometries with intramolecular hydrogen bonding and selected Natural Bond Orbital (NBO) donor–acceptor interactions of (a) poly­(2MPAEMA) and (b) 2MPAEMA-*co*-MMA.

### 3.4 Frontier Molecular Orbital (FMO) Analysis

Density-functional calculations performed on the optimized geometries^63^ show that, in the 2MPAEMA-*co*-MMA copolymer, the highest occupied molecular orbital (HOMO) is centered on the amide-phenoxy segment, whereas the lowest unoccupied molecular orbital (LUMO) is shifted toward the methacrylate carbonyls, signaling a donor-to-acceptor charge-transfer pathway upon excitation. Polymerization lengthens the conjugated backbone in poly­(2MPAEMA), allowing both frontier orbitals to spread over a wider region and narrowing the HOMO–LUMO gap from 5.207 to 4.954 eV, a change that reduces global hardness, increases softness and slightly raises the electrophilicity index. Because HOMO and LUMO energies reflect a molecule’s ability to donate or accept electrons, this gap contraction implies that the homopolymer should absorb at longer wavelengths and conduct charge more readily while retaining overall thermodynamic stability. Taken together, the frontier-orbital maps and the derived conceptual DFT descriptors indicate that poly­(2MPAEMA) exhibits enhanced electronic delocalization and intrachain charge mobility compared to its copolymer, 2MPAEMA-*co*-MMA, without compromising the intrinsic electron-withdrawing character of the backbone, making poly­(2MPAEMA) marginally more electrophilic yet still nucleophilic enough for optoelectronic or sensing uses. Figure 9 presents the Frontier Molecular Orbital analysis carried out at the B3LYP/LanL2DZ level, offering detailed insights into the electronic structures of the 2MPAEMA-*co*-MMA copolymer and the poly­(2MPAEMA) homopolymer. Table 3 highlights that polymerization from 2MPAEMA-*co*-MMA to poly­(2MPAEMA) narrows the frontier-orbital gap (Δ*E* = 5.207 → 4.954 eV) and thus lowers the global hardness (η = 2.604 → 2.477 eV) while slightly increasing the softness (σ). Concomitantly, the electronegativity (χ) and its negative counterpart, the chemical potential (μ), remain practically unchanged, indicating that the intrinsic electron-attracting tendency of the backbone is preserved during chain growth. The ionization potential (I) and electron affinity (A) were estimated from the negative of HOMO and LUMO energies, respectively (*I* = −*E*_HOMO_, *A* = −*E*_LUMO_). Using these values, we calculated several global reactivity descriptors: chemical hardness (η = (*I* – *A*)/2), softness (σ = 1/η), electronegativity (χ = (*I* + *A*)/2), chemical potential (μ = −χ), and global electrophilicity index (ω = μ^2^/2η). These values are summarized in Table 3.

**Table 3:** Calculated Quantum Chemical Descriptors for Poly­(2MPAEMA) and 2MPAEMA-co-MMA

	**values**	
**parameter**	**poly(2MPAEMA)**	**2MPAEMA-*co*-MMA**
*E*_HOMO_ (eV)	–5.837	–6.008
*E*_LUMO_ (eV)	–0.883	–0.801
Δ*E* (eV)	4.954	5.207
η (eV)	2.477	2.604
σ (eV^–1^)	0.404	0.384
χ (eV)	3.360	3.404
μ (eV)	–3.360	–3.404
ω	2.279	2.226
ε	0.920	0.855
ω^+^	1.817	1.698
ω^–^	8.537	8.507

To streamline the discussion and reduce redundancy, we integrated the results of Frontier Molecular Orbital (FMO), Molecular Electrostatic Potential (MEP), and Density of States (DOS) analyses into a summary.^64^ FMO calculations indicated a reduction in the HOMO–LUMO energy gap from 5.207 eV in the copolymer to 4.954 eV in the homopolymer, suggesting enhanced charge delocalization. MEP surface maps supported this result by showing a broader, continuous electron-rich domain in the homopolymer, favoring polar interactions. DOS profiles further confirmed greater orbital overlap and state density near the Fermi level in the homopolymer, which facilitates electronic transitions. Collectively, these findings point to improved electronic coherence and charge-transport potential in poly­(2MPAEMA), supporting its suitability for sensing and specialized optoelectronic applications such as UV photodetectors and dielectric components. In Figure 10, the frontier molecular orbital diagrams have been updated to show the energy axis labeled as Energy (eV) for HOMO and LUMO levels.

**Figure 10:**
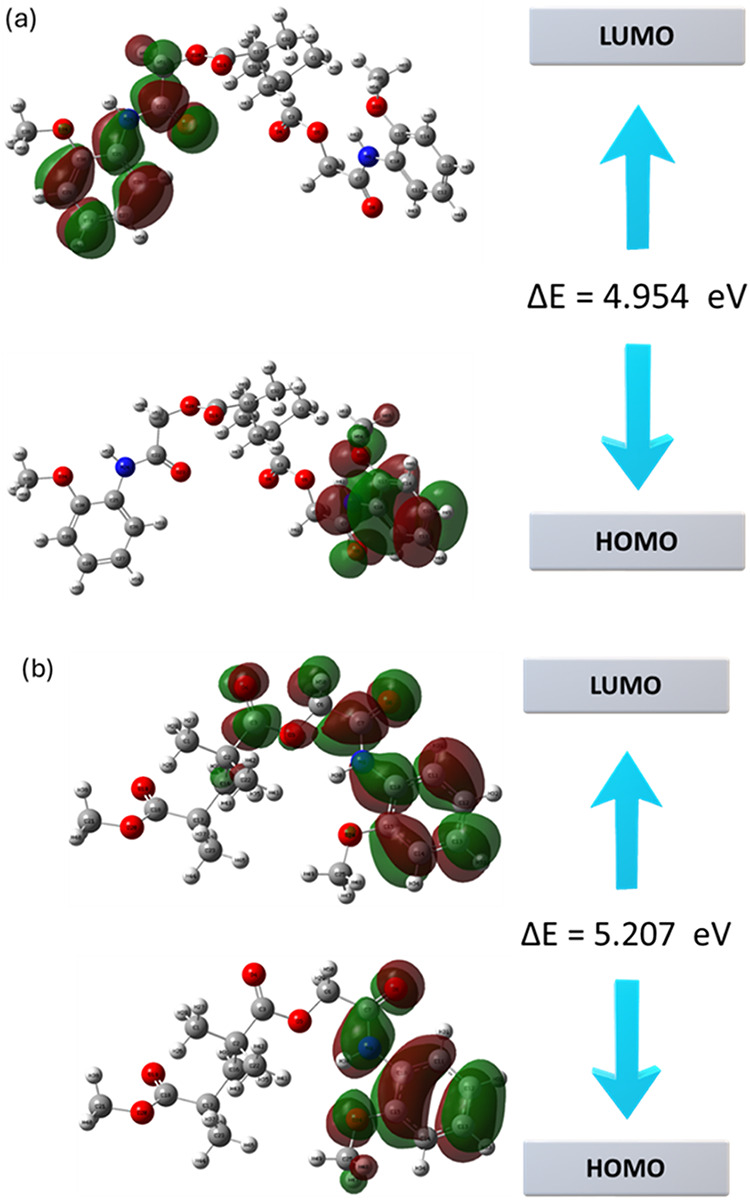
Frontier Molecular Orbital (FMO) Analysis of (a) poly­(2MPAEMA) and (b) 2MPAEMA-*co*-MMA.

Although the calculated HOMO–LUMO gap of poly­(2MPAEMA) (∼4.95 eV) falls within the range typically associated with wide-band gap insulating materials, similar values have been reported for polymers successfully applied in UV photodetectors, dielectric layers in organic electronics, and sensor matrices where charge modulation is achieved through dopants or guest–host architecture.^65–67^ For instance, poly­(methyl methacrylate) (PMMA, *E*_g_ ≈ 5.0 eV) has been employed as a dielectric interlayer to improve the performance of organic field-effect transistors and as a protective, optically active coating in photodetectors.^65^ Likewise, aromatic polyimides with gaps above 4.5 eV have demonstrated high sensitivity in humidity and gas sensors due to their polar functional groups and interfacial charge-transfer interactions.^66^ These precedents support the view that the observed wide-band gap does not preclude poly­(2MPAEMA) from optoelectronic or sensing applications, particularly when integrated into composite or hybrid device architectures.

### 3.5 Theoretical Spectroscopic Analysis

Figure 11 reveals, through FT-IR spectroscopy, the structural similarities and distinctions between poly­(2MPAEMA) and the 2MPAEMA-co-MMA copolymer. In both spectra, the medium-intensity double band at 3350–3200 cm^–1^ corresponds to secondary-amide N–H and hydrogen-bonded O–H stretches, confirming the preservation of 2MPAEMA blocks. The ester carbonyl vibration at approximately 1730 cm^–1^ appears broad and intense in poly­(2MPAEMA) but becomes sharper and more pronounced in the copolymer, unequivocally indicating the successful incorporation of methyl methacrylate (MMA) units into the chain. The weak-to-moderate band at 1650–1635 cm^–1^, characteristic of the amide carbonyl stretch, persists in the copolymer, further verifying retention of the 2MPAEMA motif. Deformation bands of methyl groups in the 1500–1385 cm^–1^ region intensify markedly in the copolymer spectrum, arising from the α-methyl groups of MMA segments. Strong, broad C–O–C stretching bands at 1260–1150 cm^–1^ in both polymers reflect ester linkages and amino-ester rings in the side chains. The absence of the expected C=C stretching band around 1630 cm^–1^ in both spectra indicates complete consumption of double bonds and near-quantitative conversion during polymerization. Figure 12 presents the ^13^C NMR spectra, which further elucidate changes in the carbon environment following polymerization. In the copolymer spectrum, many chemical shifts are observed, corresponding to diverse carbon environments including carbonyl (C=O), ester, and aliphatic carbon atoms. The MMA-derived segments exhibit distinct signals in the 10–60 ppm region, characteristic of aliphatic carbons. The spectrum of poly­(2MPAEMA) displays fewer and more closely grouped signals, reflecting a more uniform chemical environment. Figure 13, which illustrates the ^1^H NMR spectra, supports the observation of structural simplification. The streamlined appearance of the ^1^H NMR resonances signifies the emergence of a chemically uniform, more ordered polymer architecture, while the close concordance between calculated and measured FT-IR and NMR data corroborates this structural interpretation. The calculated and experimental FT-IR and NMR results align closely, demonstrating strong mutual consistency.

**Figure 11:**
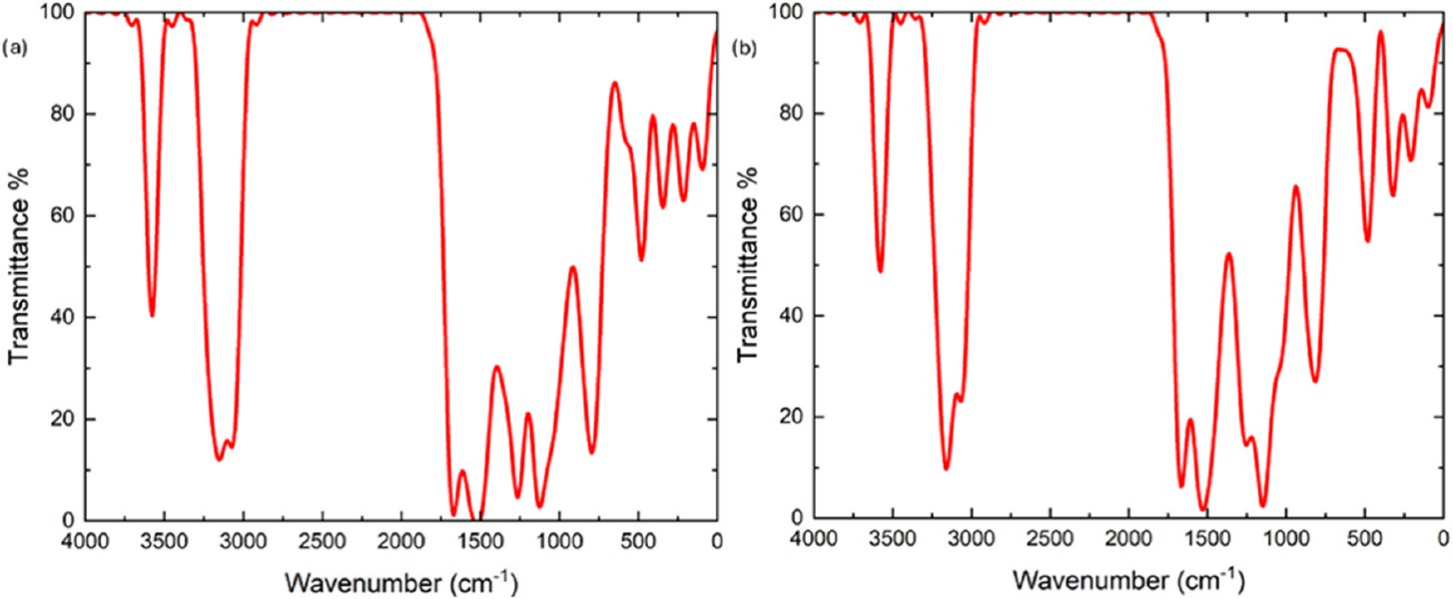
FT-IR of (a) poly­(2MPAEMA) and (b) 2MPAEMA-co-MMA.

**Figure 12:**
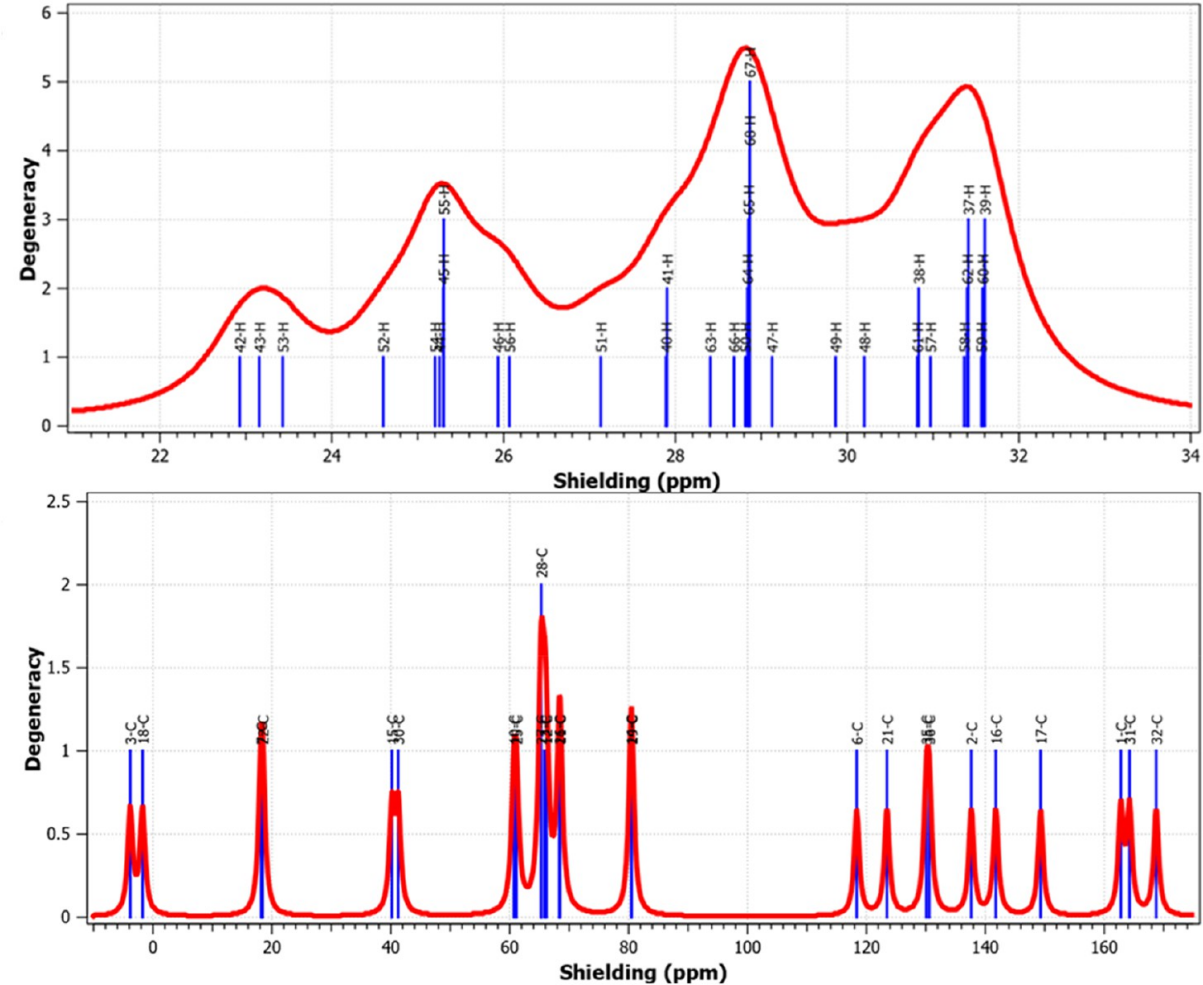
^1^H and ^13^C NMR Analysis Carbon Environment Rearrangement of poly­(2MPAEMA).

**Figure 13:**
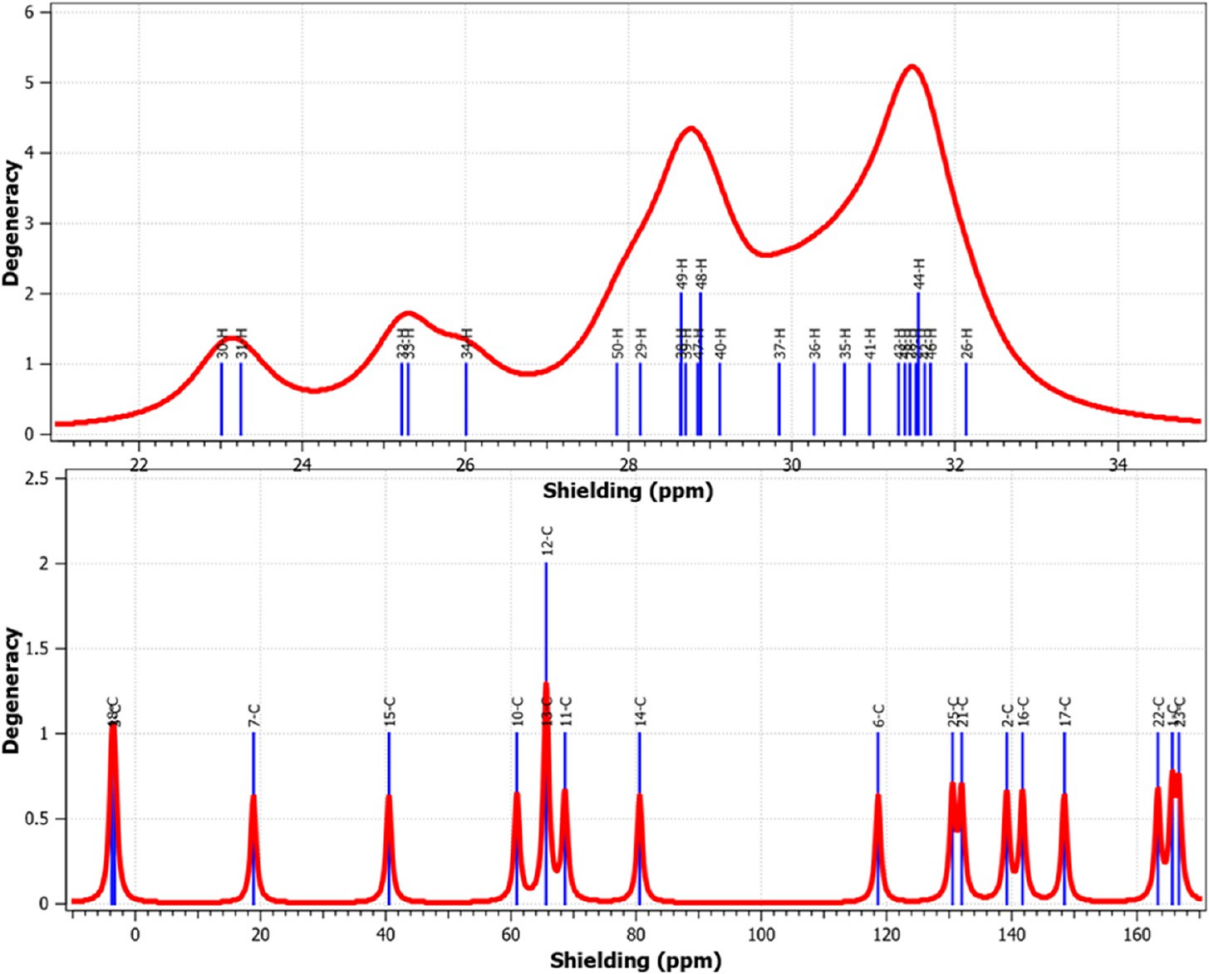
^1^H and ^13^C NMR Analysis Variation in Proton Environments of 2MPAEMA-co-MMA.

### 3.6 Molecular Electrostatic Potential (MEP)

MEP maps obtained at the DFT/B3LYP level with the LanL2DZ basis set reveal that the 2MPAEMA-co-MMA copolymer possesses an electrostatic potential distribution ranging from −6.48 × 10^–2^ to +6.48 × 10^–2^ au; the negative potential is primarily concentrated on the methacrylate carbonyl oxygens and the tertiary-amine carbonyls, whereas the positive potential is localized on the backbone α-methylene groups and the cationic ammonium nitrogens. The copolymer features nucleophilic sites at the carbonyl centers and electrophilic character at the ammonium regions, enabling strong electrostatic interactions with anionic species. The polymerized poly­(2MPAEMA) exhibits an expanded potential range of −6.90 × 10^–2^ to +6.90 × 10^–2^ au; successive carbonyl groups coalesce into a continuous electron-rich corridor, while the backbone methylene’s form a pronounced electrophilic ridge. This enhanced charge separation along the chain, by reorganizing electron density and supporting extended conjugation, may indirectly favor hydrophobic or π–π stacking interactions by enabling closer alignment of nonpolar backbone segments. The MEP surface maps in Figure 14 and Table 4 were generated at the DFT/B3LYP level with the LanL2DZ basis set and projected onto the 0.001 au electron-density isosurface.

**Figure 14:**
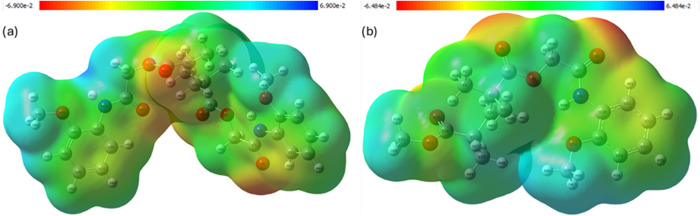
Molecular Electrostatic Potential (MEP) of (a) poly­(2MPAEMA) and (b) 2MPAEMA-*co*-MMA.

**Table 4:** Key Electron-rich Regions of (a) Poly­(2MPAEMA) and (b) 2MPAEMA-co-MMA

**system**	**color range (au)**	**key**electron-rich regions (red/orange)	**key electron-poor regions**(blue/cyan)	**implications**
**(a)**	–6.90 × 10^–2^ → +6.90 × 10^–2^	a contiguous corridor of negative potential spans successive amide-type carbonyls and pendant heteroatoms, creating an extended electron sink	backbone methylene protons and terminal methyl groups exhibit the most positive potential	polymerization amplifies charge segregation: the concatenation of carbonyls broadens the electron-rich domain, increasing the density of hydrogen-bond acceptors. Simultaneously, the hydrocarbon backbone becomes a continuous electrophilic ridge, which can promote interchain hydrophobic stacking or guest encapsulation.
**(b)**	–6.48 × 10^–2^ → +6.48 × 10^–2^	carbonyl oxygens of methacrylate and tertiary-amine carbonyls; heteroatom lone pairs	α-methylene and backbone C–H groups; quaternary-ammonium nitrogen	pronounced dipolar character favors hydrogen bonding and polar−π interactions. Carbonyl centers are the most probable loci for nucleophilic attack, while the cationic ammonium site presents an electrophilic hot-spot, rationalizing the copolymer’s affinity for anionic substrates.

### 3.7 Theoretical Thermochemistry Surface Maps (TCSM)

Figure 15 depicts the TCSM generated at the B3LYP/LanL2DZ level for (a) 2MPAEMA-*co*-MMA and (b) poly­(2MPAEMA). In the copolymer, the most stabilizing (low-energy) domains colored deep red are confined to the ester and amide carbonyl oxygens, whereas destabilizing (high-energy, yellow-orange) regions cluster around the tertiary-amine nitrogen and the neighboring α-methylene units. After polymerization, these isolated red pockets coalesce into a continuous, electron-rich corridor that extends along successive carbonyl groups, while the hydrocarbon backbone evolves into a broad, thermally labile ridge. Hence, poly­(2MPAEMA) combines an extended (thermal sink) that can dissipate vibrational energy with a flexible backbone capable of accommodating conformational changes. Linear-fit slopes (300–900 K) quantify the steeper temperature response of the homopolymer for *C*_V_ and *S*; see Figure 16. At every temperature, poly­(2MPAEMA) displays higher values of *E*thermal, *C*_v_, and *S* than the copolymer, reflecting its larger number of vibrational degrees of freedom and greater configurational flexibility. The steeper slopes observed for the homopolymer particularly in *C*_v_ (≈ 25% above the copolymer) and entropy (an additional 40 J mol^–1^ K^–1^ at 900 K) indicate superior thermal buffering capacity and a stronger tendency toward disorder. Collectively, these thermochemical trends suggest that polymerization enhances heat-storage ability, promotes solvation and hydrogel formation, and endows the material with cooperative binding sites that could be exploited in temperature-responsive or adsorption-based applications. TCSM exhibit excellent agreement with the experimentally determined thermal profiles of both the homopolymer and the copolymer. These results predict that the copolymer segments stiffen the polymer chains, increasing the degradation threshold, while the homopolymer absorbs energy more effectively against temperature increases due to its higher thermal buffering capacity. The experimental and theoretical findings consistently explain the thermal performance profiles of the materials.

**Figure 15:**
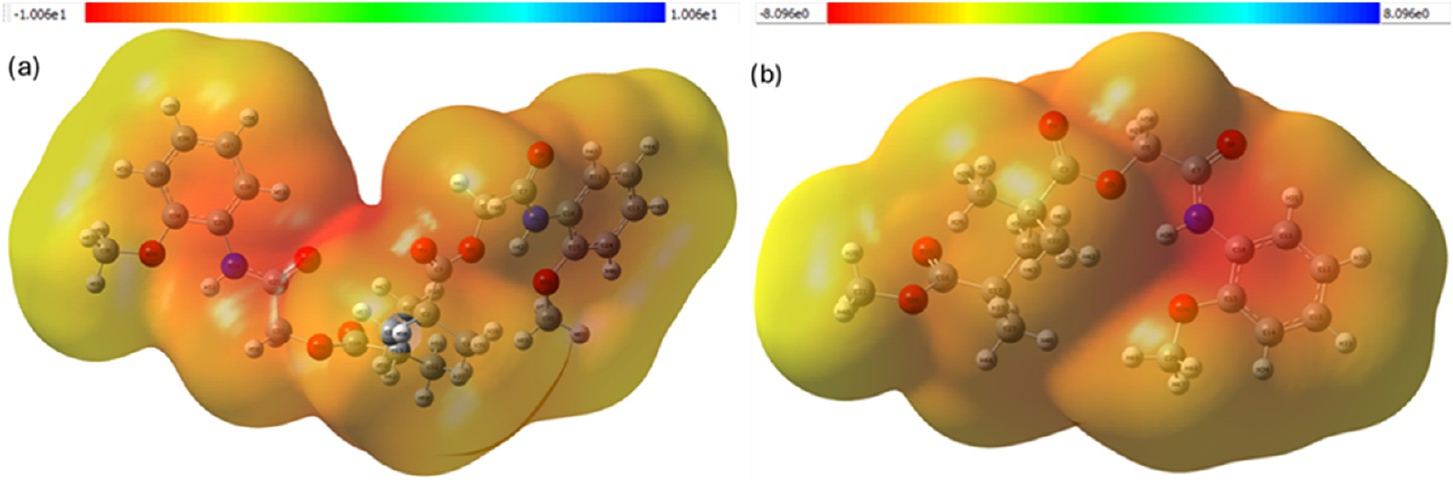
Thermochemistry Surface Maps (TCSM) for (a) poly­(2MPAEMA) and (b) 2MPAEMA-*co*-MMA projected on the 0.001 au isosurface at 298.15 K and 1 atm (RRHO). The color bar shows the dimensionless local thermochemical index (0 = highly stabilizing/low local thermal-energy density, deep-red; 1 = less stabilizing/high local thermal-energy density, yellow/orange).

**Figure 16:**
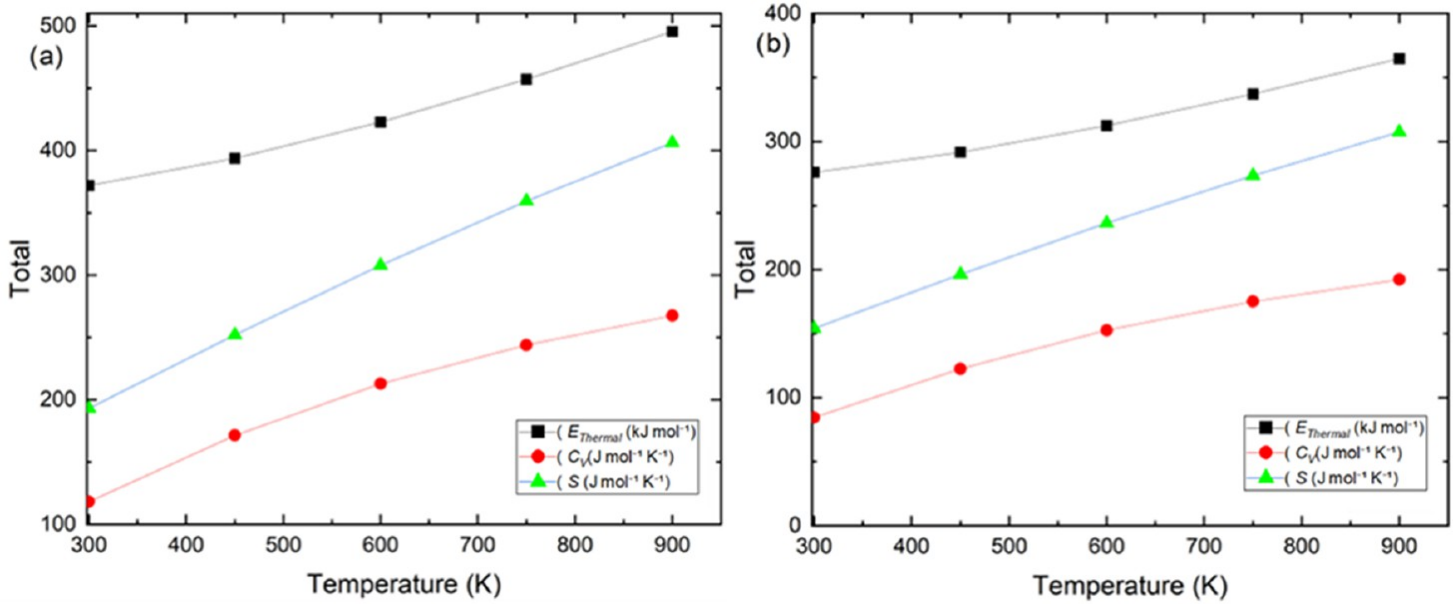
Temperature dependence of molar thermal energy (*E*_thermal_), constant-volume heat capacity (*C*_V_), and entropy (*S*) for poly­(2MPAEMA) and 2MPAEMA-*co*-MMA (RRHO, 1 atm; 300–900 K). Units: *E*_*thermal*_ (kJ mol^–1^), *C*_V_ (J mol^–1^ K^–1^), *S* (J mol^–1^ K^–1^).

### 3.8 Density of States (DOS)

Figure 17 and Table 5 compare the total electronic density of states for (a) 2MPAEMA-*co*-MMA and (b) poly­(2MPAEMA), calculated at the B3LYP/LanL2DZ level and referenced to the vacuum-level energy axis (0 eV set at the top of the valence manifold). Green ticks mark occupied molecular orbitals, red ticks denote virtual (unoccupied) orbitals, and the blue trace represents the Gaussian-broadened DOS profile. The density-of-states (DOS) data obtained at the B3LYP/LanL2DZ level indicate that, while the 2MPAEMA-*co*-MMA copolymer possesses a HOMO–LUMO gap of about 4.2 eV and only a modest density of edge states, polymerization to poly­(2MPAEMA) introduces additional σ/π interactions that broaden the valence-bandwidth by roughly 1 eV, narrow the gap to ∼ 3.6 eV, and elevate the state density near the Fermi level, thereby enabling lower-energy optoelectronic transitions and enhanced charge-transport potential.

**Figure 17:**
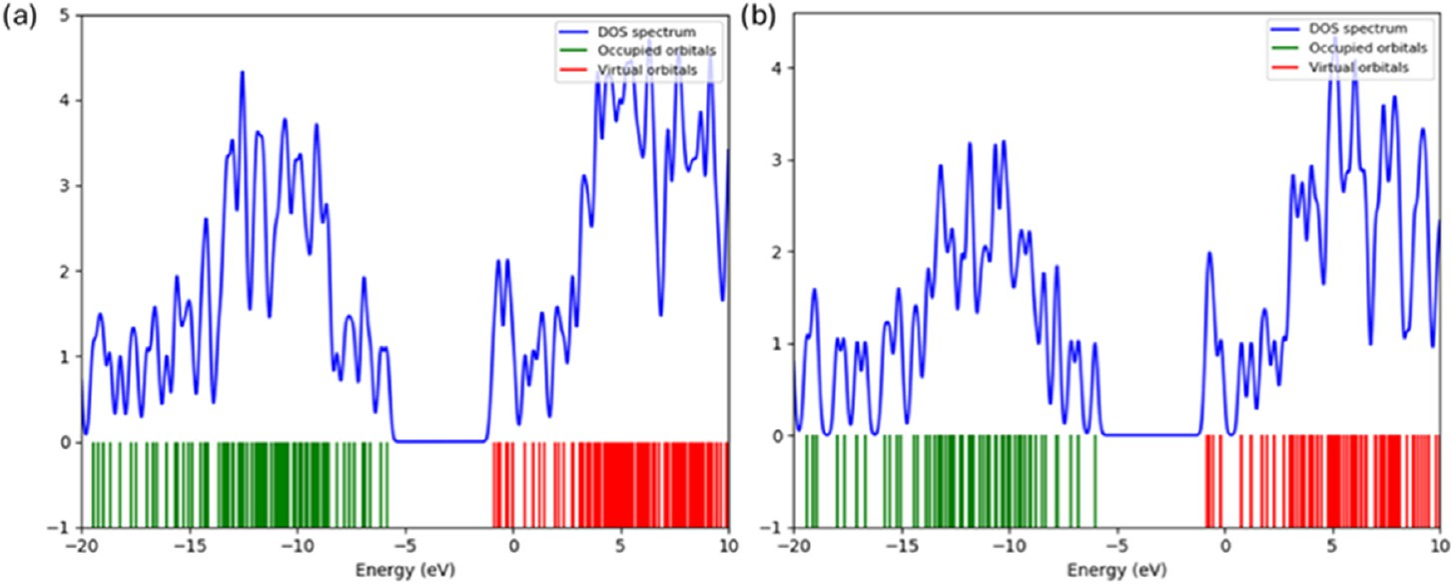
Density of States (DOS) of (a) poly­(2MPAEMA) and (b) 2MPAEMA-*co*-MMA.

**Table 5:** Compares the Total Electronic Density of States for Poly­(2MPAEMA) and 2MPAEMA-co-MMA

**parameter**	**poly(2MPAEMA)**	**2MPAEMA-co-MMA**	**consult**
**valence-bandwidth** (occupied states)	slightly wider (∼15 eV)	∼14 eV (−19 → −5 eV)	polymerization introduces additional σ/π combinations, broadening the occupied manifold by ≈1 eV.
**conduction-band onset** (first virtual state)	≈+0.8 eV	≈+1.2 eV	a 0.4 eV down-shift signals better electronic delocalization in the homopolymer.
**HOMO–LUMO gap** (Δ*E*_gap_)	≈3.6 eV	≈4.2 eV	polymerization narrows the gap by ∼ 0.6 eV, consistent with enhanced conjugation and increased chain length.
**DOS intensity near band edges**	higher DOS within ±2 eV of Fermi level	moderate peaks at –5 → −4 eV and +2 → +4 eV	a denser state population close to the frontier orbitals should facilitate charge transport and lower excitation energies.

### 3.9 Non-Covalent Interactions (NCI)

The NCI is surfaces in Figure 18 reveal that, in the 2MPAEMA-co-MMA copolymer, green regions corresponding to weak van der Waals contacts and a few blue patches indicative of moderate hydrogen bonds cluster mainly around the carbonyl oxygens. Along the poly­(2MPAEMA) chain these green domains merge into a continuous band, while the expanded blue-green areas point to more pronounced intramolecular hydrogen bonding and π-stacking interactions. The RDG scatter plots in Figure 19 show a dense cloud of green points near sign­(λ_2_)­ρ ≈ 0 for both systems, confirming the prevalence of weak dispersive forces, whereas negative (blue) regions mark attractive hydrogen bonds. For poly­(2MPAEMA) the distribution spreads farther into both negative and positive regions, indicating that polymerization concurrently amplifies van der Waals and steric (red) interactions, thereby enhancing chain flexibility and conformational diversity. To ensure clarity across both spatial (Figure 18) and scatter (Figure 19) NCI representations, we confirm that in both figures, blue signifies attractive interactions such as hydrogen bonding, while red indicates repulsive (steric) contacts. For poly­(2MPAEMA), the RDG distribution extends further into both the negative and positive sign­(λ_2_)­ρ regions, signifying a broader spectrum of noncovalent interactions. This is reflected in stronger intramolecular hydrogen bonding (blue) and steric repulsion (red), which together contribute to the polymer’s conformational adaptability.

**Figure 18:**
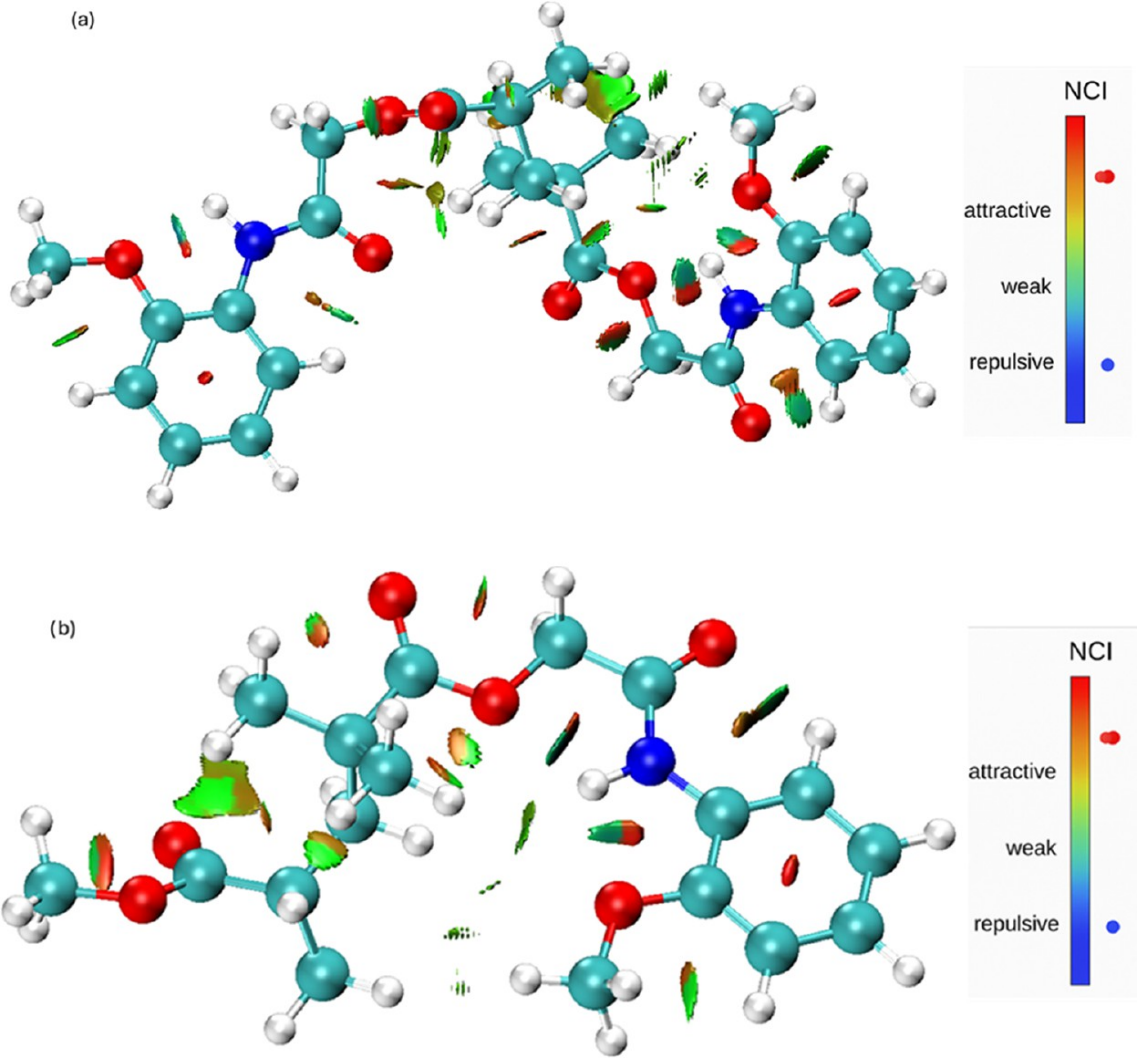
NCI of (a) poly­(2MPAEMA) and (b) 2MPAEMA-co-MMA.

**Figure 19:**
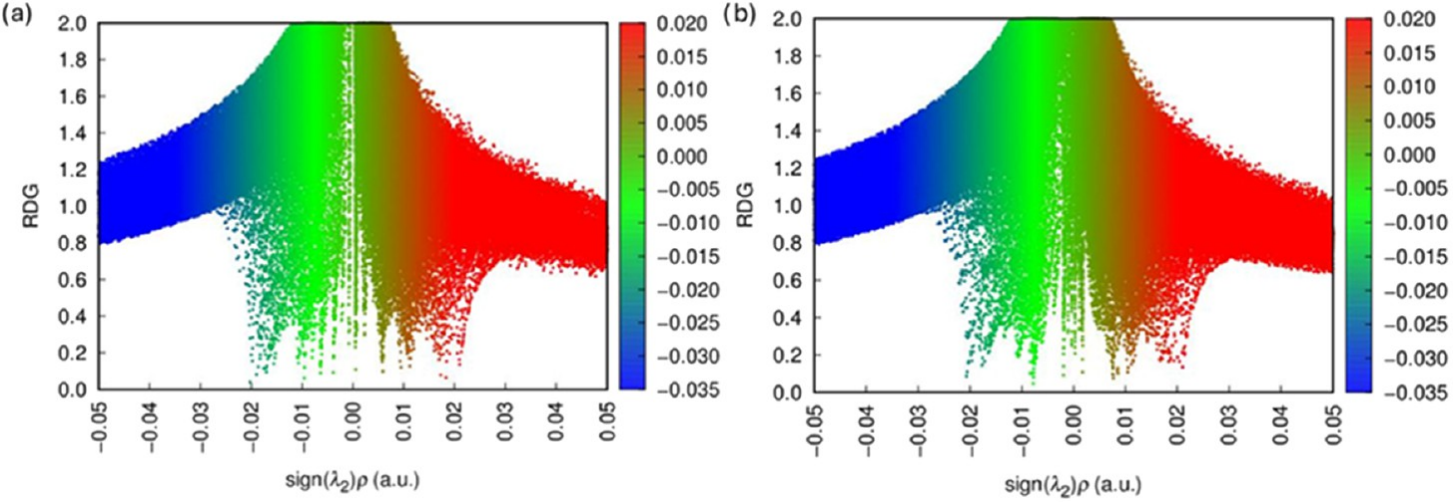
RDG of (a) poly­(2MPAEMA) and (b) 2MPAEMA-co-MMA.

## 4 Conclusions

This study provides a comprehensive characterization of polymeric derivatives of 2-(2-methoxyphenylamino)-2-oxoethyl methacrylate (2MPAEMA), integrating experimental and theoretical insights to reveal key structure–property relationships. The close agreement between DFT-based analyses (MEP, NBO, DOS, thermochemistry) and experimental data (FT-IR, NMR, TGA/DTA/DTG) confirms the reliability of the applied computational protocol (B3LYP/LanL2DZ) for predicting the structural, electronic, and thermal properties of methacrylate-based polymers. Both the homopolymer (poly­(2MPAEMA)) and the copolymer (2MPAEMA-co-MMA) were successfully synthesized via free radical polymerization and structurally verified by spectroscopy. DFT calculations indicated that polymer geometries represent true energy minima, while frontier orbital and DOS analyses highlighted a narrower HOMO–LUMO gap, stronger orbital delocalization, and enhanced intramolecular cohesion in the homopolymer, suggesting improved charge transport and photophysical properties. Although the calculated HOMO–LUMO gap of poly­(2MPAEMA) (∼4.9 eV) indicates a wide-band gap character, such values do not preclude potential use in UV photodetection, dielectric materials in organic electronics, or insulating matrices in composite sensors. Recent studies have shown that high-gap polymers can exhibit notable sensing performance when combined with charge-modulating guest interactions or field-responsive architectures. Thermal analysis further demonstrated robust stability, supported by theoretical thermochemical mapping. Collectively, these findings validate the structural integrity, electronic tunability, and thermal robustness of 2MPAEMA-based polymers, positioning them as promising candidates for optoelectronic devices, biosensors, and smart coatings. Looking forward, future studies will focus on tailoring these materials for specific uses such as drug delivery, antifouling coatings, and biological sensing, while also evaluating their environmental stability and biocompatibility. This forward-looking research direction emphasizes their versatility and practical potential across advanced functional materials.

## Data Availability

The data underlying this article will be shared on reasonable request to the corresponding author.
